# Does Robotic Adrenalectomy Outperform Laparoscopic Approaches in Obese Patients? A Systematic Review and Subgroup Meta‐Analysis of 1,107 Patients

**DOI:** 10.1002/rcs.70169

**Published:** 2026-04-20

**Authors:** Ahmed Abdelsamad, Youssef Badie, Mohamed Elfakharany, Mohamed Elatrosh, Mohamed Elgohary, Eslam Elmaghraby, Florian Gebauer, Ralf Mike Langenbach, Khaled Mohamed

**Affiliations:** ^1^ Department of Surgery II University of Witten/Herdecke Witten Germany; ^2^ Deputy Head of the Oncological Surgery Department Section Head of Robotic Surgery Knappschaft Vest‐Hospital Recklinghausen Germany; ^3^ Faculty of Medicine Alexandria University Alexandria Egypt; ^4^ Core Surgical Trainee Yorkshire and The Humber Hull UK; ^5^ Faculty of Medicine Cairo University Cairo Egypt; ^6^ Faculty of Medicine New Giza University Cairo Egypt; ^7^ Department of Surgery University Hospital Düsseldorf ‐UKD Düsseldorf Germany; ^8^ Department of Surgery University Hospital Helios Wuppertal Wuppertal Germany; ^9^ General Surgery and GIT Surgery Head Klinikum Lippstadt Lippstadt Germany

**Keywords:** laparoscopic adrenalectomy, meta‐analysis, minimally invasive surgery, obesity, robotic adrenalectomy

## Abstract

**Background:**

Obesity poses technical challenges in adrenalectomy. Robotic adrenalectomy (RA) offers advantages over laparoscopic adrenalectomy (LA), but evidence in obese patients remains limited.

**Methods:**

A meta‐analysis of comparative studies through 2025 assessed perioperative outcomes of RA versus LA, with subgroup analyses by body habitus (obese vs. non‐obese) and surgical approach (lateral transabdominal [LT] vs. posterior retroperitoneal [PR]). Primary outcomes included operative time (OT), blood loss (EBL), hospital stay (LOHS), complications, conversion, and mortality. Study quality was evaluated using the Newcastle–Ottawa Scale, and certainty was assessed using the GRADE approach.

**Results:**

Eight studies (1107 patients) were included. RA was associated with reduced EBL (*p* < 0.001) and shorter LOHS (*p* < 0.001), with no other significant differences. Subgroup analysis showed shorter LOHS in non‐obese LA patients (*p* = 0.03). PR showed a shorter LOHS (*p* = 0.001) compared with LT.

**Conclusions:**

RA provides perioperative benefits, particularly reduced blood loss and shorter hospital stay, without compromising safety.

**Abbreviations**
BMI: body mass index
EBL: estimated blood loss
LA: laparoscopic adrenalectomy
LOHS: length of hospital stay
LT: lateral transabdominal
OT: operative time
PR: posterior retroperitoneal
RA: robotic adrenalectomy

## 1 Introduction

Adrenal tumors are relatively uncommon, with an estimated prevalence ranging from 1% to 5% in the general population, typically discovered incidentally during imaging for unrelated conditions [1]. Although most adrenal masses are benign and nonfunctional, a subset requires surgical intervention due to hormonal hypersecretion, malignancy risk, or size progression [2]. Indications for adrenalectomy include functioning tumors such as pheochromocytomas, aldosteronomas, and cortisol‐producing adenomas, as well as nonfunctioning masses suspected to be malignant or larger than 4–6 cm [3, 4].

Advances in surgical techniques have significantly impacted the approach to adrenalectomy. Traditional open surgery has largely been replaced by minimally invasive techniques, primarily laparoscopic adrenalectomy (LA), which has become the standard approach due to its favorable outcomes in terms of postoperative recovery and complication rates [5, 6]. More recently, robotic‐assisted adrenalectomy (RA) has gained popularity, offering enhanced dexterity, 3D visualization, and ergonomic advantages [7, 8]. In parallel, different anatomical access routes have emerged—namely, the transabdominal lateral (LT) and posterior retroperitoneal (PR) approaches—each with specific advantages based on tumor characteristics, surgeon expertise, and patient body habitus [9].

Recent experimental work has expanded the understanding of adrenal‐related tumor biology by developing succinate dehydrogenase subunit B (SDHB)‐deficient tumor models, which may serve as valuable platforms for future preclinical therapeutic and immunotherapy research, as demonstrated by Hadrava Vanova et al. (2025) [10].

Obesity is a growing global health issue and poses technical challenges during minimally invasive adrenalectomy [11]. However, the effect of obesity on surgical outcomes in adrenalectomy remains controversial [12]. While some studies suggest increased operative complexity and complication rates in obese patients [13, 14], others report comparable outcomes [15, 16].

Despite the increasing adoption of robotic techniques and evolving access strategies, no prior systematic review and meta‐analysis (SRMA) has comprehensively compared robotic versus laparoscopic adrenalectomy and lateral versus posterior approaches, with subgroup analyses focused on obese versus non‐obese patients. Furthermore, evidence evaluating perioperative outcomes stratified by surgical modality and patient body mass index remains scarce.

This systematic review and meta‐analysis aim to fill this knowledge gap by comparing robotic and laparoscopic adrenalectomy across both lateral transabdominal and posterior retroperitoneal approaches. Additionally, it investigates differences in perioperative outcomes between obese and non‐obese patients undergoing minimally invasive adrenalectomy to inform surgical decision‐making and optimize patient selection for various minimally invasive adrenalectomy techniques.

## 2 Materials and Methods

### 2.1 Ethical Approval

The protocol for this systematic review was prospectively registered in the PROSPERO database under the reference number **CRD420251102588**, ensuring transparency and minimizing reporting bias. All included studies had obtained prior institutional review board (IRB) or ethical approval. The study was meticulously conducted following the methodological standards outlined by the Cochrane Collaboration and reported in compliance with the Preferred Reporting Items for Systematic Reviews and Meta‐Analyses (PRISMA) guidelines [17, 18]. A complete PRISMA checklist is provided in Supporting Information Table S1. In addition, the manuscript was designed and structured to adhere to the **AMSTAR 2 tool**, which allows assessment of the methodological quality of systematic reviews (Supporting Information Table S2) [19].

### 2.2 Search Strategy

**FIGURE 1:**
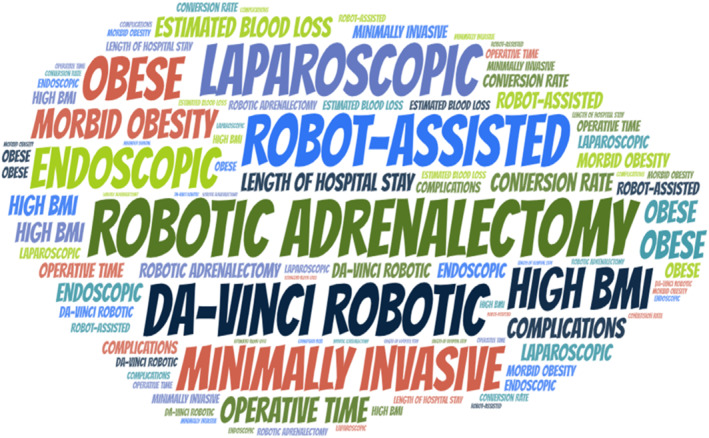
Word cloud of the MeSH terms. Visual representation of the frequency and distribution of key search terms used in the literature search strategy.

A comprehensive literature search strategy was implemented across three major electronic databases—PubMed, Scopus, and Web of Science—with coverage of all published literature up to January 7, 2025. The search was deliberately structured to capture all relevant studies that investigated outcomes of adrenalectomy in obese patients as defined by the World Health Organization (WHO) classification (BMI ≥ 30). The intervention of interest was robotic adrenalectomy (RA), which was compared with laparoscopic adrenalectomy (LA) in patients undergoing surgery for adrenal tumors. To ensure inclusivity and maximize sensitivity, the search incorporated a wide spectrum of keywords and synonyms related to robotic adrenal surgery (including “Robotic adrenalectomy,” “Robot‐assisted adrenalectomy,” “Da Vinci robotic adrenalectomy,” “Robotic adrenal surgery,” “Robotically assisted adrenalectomy,” and “Robot‐enhanced adrenalectomy”), in combination with laparoscopic or minimally invasive terminology (“Laparoscopic adrenalectomy,” “Minimally invasive adrenalectomy,” “Endoscopic adrenalectomy,” “Laparoscopic adrenal surgery,” and “Keyhole adrenal surgery”). Furthermore, obesity‐related terms were included to guarantee that the target population was identified, including “Obese,” “High body mass index,” “High BMI,” “Overweight,” “Morbid obesity,” and “Super obese.” The **word cloud** (Figure 1) illustrates the distribution and frequency of these terms across the retrieved records, reflecting the comprehensiveness of the search process.

### 2.3 Study Design and Outcomes (PICOS Framework)

The study was designed within the framework of **PICOS criteria (Population, Intervention, Comparator, Outcomes, Study design)**. The **population** (P) included obese patients (BMI ≥ 30) undergoing adrenalectomy for adrenal tumors. The **intervention** (I) consisted of robotic adrenalectomy, while the **comparison** (C) group consisted of laparoscopic adrenalectomy. The **primary outcomes** (O) were defined as operative time and estimated blood loss, given their relevance to perioperative safety and efficiency. **Secondary outcomes** included transfusion rate, intraoperative hemodynamic instability, conversion to open surgery, length of hospital stay, and overall complication rate. The **study designs (S)** eligible for inclusion encompassed randomized controlled trials (RCTs), prospective or retrospective cohort studies, and case–control studies, provided they offered comparative data between robotic and laparoscopic adrenalectomy in obese patients. This strict adherence to the PICOS criteria allowed systematic evaluation of clinically relevant endpoints while maintaining methodological rigor.

### 2.4 Study Selection and Screening

Eligibility was strictly applied to include human studies only, with the exclusion of case reports, reviews, animal experiments, and studies focusing exclusively on non‐obese populations or those not including robotic adrenalectomy. Following the database search, all identified records were imported into EndNote X9 software for systematic organization and de‐duplication. Duplicates were removed electronically, after which the dataset was exported into Microsoft Excel for further processing. A two‐stage screening process was performed: first, titles and abstracts were reviewed to identify potentially relevant studies; second, full‐text assessments were conducted to determine eligibility based on predefined inclusion and exclusion criteria. Each stage of the screening was independently carried out by three reviewers, ensuring objectivity and minimizing bias. Any discrepancies or disagreements were resolved by a fourth senior reviewer, thereby strengthening the reliability of the selection process.

### 2.5 Data Extraction and Outcomes

For data extraction, four independent reviewers worked in parallel to collect standardized information from each eligible study using a predesigned Excel spreadsheet. Extracted information included study characteristics (first author, year of publication, country, and study design; summarized in Table 1) as well as baseline characteristics of the study populations (mean age, sex distribution, body mass index, tumor laterality, tumor size, and pathological diagnosis; summarized in Table 2). Furthermore, data comparing outcomes in obese versus non‐obese cohorts within the included studies were separately extracted and summarized in Table 3. The surgical outcomes of interest were operative time, estimated blood loss, conversion rate, hospital stay, complications, transfusion requirements, intraoperative instability, and perioperative mortality. Disagreements during the data collection stage were resolved by group discussion and consensus, ensuring the accuracy and completeness of the extracted information.

**TABLE 1:** Study characteristics. Characteristics of the included studies, including year, country, study design, and surgical approach.

Study and year	Country	Study type	Technique/Approach
Aksoy (2013) [20]	USA	Retrospective cohort	Lateral transabdominal approach, posterior retroperitoneal approach
Isiktas (2023) [21]	USA	Retrospective cohort	Lateral transabdominal approach, posterior retroperitoneal approach
Shalaby (2021) [22]	USA	Retrospective cohort	Lateral transabdominal approach, posterior retroperitoneal approach
De Crea (2022) [23]	Italy	Retrospective cohort	Lateral transabdominal approach
Erdemir (2022) [24]	Turkey	Retrospective cohort	Lateral transperitoneal approach
Verhoeff (2025) [25]	Italy	Retrospective cohort	Lateral transabdominal approach, posterior retroperitoneal approach
Knewitz (2024) [26]	USA	Retrospective cohort	—
Agcaoglu (2019) [27]	Turkey	Retrospective cohort	Lateral transperitoneal approach

**TABLE 2:** Baseline characteristics of obese patients undergoing robotics versus laparoscopic adrenalectomy. Demographic, anthropometric, and tumor‐related characteristics of obese patients undergoing robotic adrenalectomy (RA) or laparoscopic adrenalectomy (LA).

Study	Study groups	Number of patients	Age (mean, SD)	Sex (F/M)	BMI (mean, SD)	Tumor size (mean, SD)	Tumor side (left/Right/Bilateral)	Perirenal fat thickness (mean, SD)	Skin to Gerota's fascia distance (mean, SD)	Diagnosis					
										Cushing's	Non‐secreting adrenocortical adenoma	Primary hyperaldosteronism	Pheochromocytoma	Malignancy	Others
Aksoy (2013) [20]	Robotic	42	54.2 ± 2	29\13	35.4 ± 1	4 ± 0.4	32\21\4	N/A	N/A	10	10	6	8	0	8
	Laparoscopic	57	51.3 ± 1.7	35\22	38.8 ± 0.8	4.3 ± 0.3	21\20\1	N/A		10	16	8	12	3	8
Isiktas (2023) [21]	Robotic	94	50.1 ± 16.56	71\20	41.5 ± 4.74	36.1 ± 22.59	56\38\0	18.1 ± 9.64	86.7 ± 20.18	37	17	19	0	5	16
	Laparoscopic	67	50 ± 15.91	40\23	42.4 ± 6.97	42.1 ± 24.24	43\24\0	19.4 ± 16.29	88.5 ± 23.49	21	16	9	0	3	18
Knewitz (2024) [26]	Robotic	69	N/A	N/A	N/A	N/A	N/A	N/A	N/A	N/A	N/A	N/A	N/A	N/A	N/A
	Laparoscopic	69	N/A	N/A	N/A	N/A	N/A	N/A	N/A	N/A	N/A	N/A	N/A	N/A	N/A
Shalaby (2021) [22]	Robotic	41	53.07 ± 13.22	20\21	37.48 ± 6.26	4.17 ± 2.41	19\22\0	N/A	N/A	N/A	N/A	N/A	N/A	N/A	N/A
	Laparoscopic	14	57.43 ± 11.93	7\7	35.59 ± 4.27	4.81 ± 4.74	8\6\0	N/A	N/A	N/A	N/A	N/A	N/A	N/A	N/A
De Crea (2022) [23]	Robotic	39	N/A	N/A	N/A	N/A	N/A	N/A	N/A	N/A	N/A	N/A	N/A	N/A	N/A
	Laparoscopic	39	N/A	N/A	N/A	N/A	N/A	N/A	N/A	N/A	N/A	N/A	N/A	N/A	N/A
Verhoeff (2025) [25]	Robotic	45	53.87 ± 13.73	N/A	32.07 ± 1.51	3.94 ± 2.13	N/A	N/A	N/A	0	0	0	45	0	0
	Laparoscopic	531	51.88 ± 14.51	N/A	33.68 ± 4.14	4.24 ± 2.09	N/A	N/A	N/A	0	0	0	531	0	0

**TABLE 3:** Baseline characteristics of obese versus non‐obese patients. Demographics, anthropometric, and tumor‐related characteristics of obese and non‐obese patients stratified using surgical approach.

Study	Approach	Number		Age/years		Gender (F/M)		BMI (kg/m^2^)		Tumor side (L/R/B)		Tumor size/mm		Diagnosis									
		Obese	Nonobese	Obese	Nonobese	Obese	Nonobese	Obese	Nonobese	Obese	Nonobese	Obese	Nonobese	Cushing's		Nonsecreting adrenocortical adenoma		Pheochromocytoma		Primary hyperaldosteronism		Malignancy	
														Obese	Nonobese	Obese	Nonobese	Obese	Nonobese	Obese	Nonobese	Obese	Nonobese
Shalaby (2021) [22]	Laparoscopic	41	29	57.43 ± 11.93	55.28 ± 15.29	7/7	18/11	35.59 ± 4.27	26.58 ± 1.85	8/6/0	24/5/0	48.1 ± 47.4	30.7 ± 19.5	N	N	N	N	N	N	N	N	N	N
	Robotic	14	36	53.07 ± 13.22	58.50 ± 12.81	20/21	17/19	37.48 ± 6.26	24.72 ± 4.11	19/22/0	18/18/0	41.7 ± 24.1	40.9 ± 23.2	N	N	N	N	N	N	N	N	N	N
De Crea (2022) [23]	Laparoscopic	39	53	N/A	N/A	N/A	N/A	N/A	N/A	N/A	N/A	N/A	N/A	N/A	N/A	N/A	N/A	N/A	N/A	N/A	N/A	N/A	N/A
	Robotic	39	53	N/A	N/A	N/A	N/A	N/A	N/A	N/A	N/A	N/A	N/A	N/A	N/A	N/A	N/A	N/A	N/A	N/A	N/A	N/A	N/A
Agcaoglu (2019) [27]	Robotic	26	40	49 ± 13	46 ± 14	18/8	22/18	38.25 ± 6.81	24.75 ± 2.55	11/15/0	23/17/0	41 ± 26	45 ± 29	3	6	8	14	10	14	3	4	2	2
Erdemir (2022) [24]	Robotic	12	18	N/A	N/A	N/A	N/A	N/A	N/A	N/A	N/A	N/A	N/A	N/A	N/A	N/A	N/A	N/A	N/A	N/A	N/A	N/A	N/A

### 2.6 Quality Assessment

The methodological quality of the included studies was critically evaluated using the Newcastle–Ottawa Scale (NOS), which assesses observational studies across three domains: (i) the adequacy of selection of study groups, (ii) comparability of groups, and (iii) ascertainment of exposure/outcome. A maximum of nine points could be awarded per study, with scores of 7–9 classified as high quality, 4–6 as moderate quality, and 0–3 as low quality. Quality assessment was independently performed by two reviewers, and any discordance in scoring was discussed and adjudicated by a third reviewer. The summary of NOS scoring is presented in Table 4, demonstrating that most studies included in this meta‐analysis were of high or moderate quality, thereby supporting the robustness of the evidence base.

**TABLE 4:** Newcastle–Ottawa scale quality assessment of included studies. Methodological quality assessment of the included observational studies using the Newcastle–Ottawa scale.

Study	Selection maximum: ★★★★				Comparability maximum: ★★	Outcome maximum: ★★★			Total stars (out of 9)
	Representativeness maximum: ★	Non‐exposed cohort maximum: ★	Exposure ascertainment maximum: ★	Outcome absent at start maximum: ⋆	Comparability	Assessment	Follow‐up long enough	Adequacy of follow‐up	
Aksoy et al. (2013) [20]	★	★	★		★★	★		★	7
Agcaoglu et al. (2019) [27]	★	★	★	★	★	★	★	★	8
Shalaby et al. (2021) [22]	★	★	★	★	★	★	★	★	8
Isiktas et al. (2023) [21]	★	★	★	★	★★	★	★	★	9
Erdemir et al. (2022) [24]	★		★	★		★	★	★	6
De Crea et al. (2022) [23]	★	★	★	★	★★	★	★	★	9
Verhoeff et al. (2024) [25]	★	★	★	★	★★	★	★	★	9

*Note:* Newcastle‐Ottawa Scale (NOS) Quality Assessment Table.

### 2.7 Certainty of Evidence

In parallel, the certainty of evidence for each outcome was graded using the GRADE (Grading of Recommendations, Assessment, Development and Evaluation) framework. This approach considers five key domains—risk of bias, inconsistency, indirectness, imprecision, and publication bias—to determine whether the level of confidence in the estimated effects should be rated as high, moderate, low, or very low. GRADE assessments were performed independently by two reviewers, with consensus achieved for final judgments.

### 2.8 Statistical Analysis

All statistical analyses were conducted using Review Manager (RevMan) version 5.4.1. [28]. For continuous variables, including operative time, estimated blood loss, and hospital stay, data were extracted as means with standard deviations (SD). In cases where studies reported medians with interquartile ranges, values were transformed into approximate means and SDs using validated statistical methods. Mean differences (MDs) with corresponding 95% confidence intervals (CIs) were calculated for each continuous endpoint [29]. For dichotomous variables, including complication rates, transfusion requirements, conversion to open surgery, intraoperative instability, and mortality, odds ratios (ORs) with 95% CIs were computed.

Given the expected clinical and methodological heterogeneity across the included observational studies, a random‐effects model based on the DerSimonian and Laird method was applied to pool effect estimates [30]. Statistical heterogeneity was assessed using the Cochran's *Q* test and quantified by the *I* ^2^ statistic, with thresholds of 25%, 50%, and 75% representing low, moderate, and high heterogeneity, respectively. A *p*‐value of < 0.05 was considered to indicate statistical significance.

To further enhance the robustness of the findings, subgroup analyses were prespecified. First, comparative outcomes of RA versus LA were separately analyzed in obese patients to determine whether robotic platforms confer specific advantages in this high‐risk population. Second, outcomes of obese versus non‐obese individuals were stratified by minimally invasive surgical modality (robotic vs. laparoscopic). Third, a detailed subgroup comparison was conducted according to the surgical approach—lateral transabdominal (LT) versus posterior retroperitoneal (PR)—with results stratified by robotic, laparoscopic, or combined modality, depending on reported outcomes within each study.

Furthermore, sensitivity analyses were performed using the leave‐one‐out method, sequentially excluding each study from the pooled analysis to assess its influence on overall estimates and to identify potential outliers driving heterogeneity. The consistency of results across sensitivity analyses was used to strengthen confidence in the conclusions drawn from the meta‐analysis.

By integrating rigorous methodology, adherence to international reporting standards, comprehensive subgroup analyses, and robust statistical methods, this systematic review and meta‐analysis aimed to provide the most reliable and clinically relevant evidence on the comparative safety and efficacy of robotic versus laparoscopic adrenalectomy in obese patients.

## 3 Results

**FIGURE 2:**
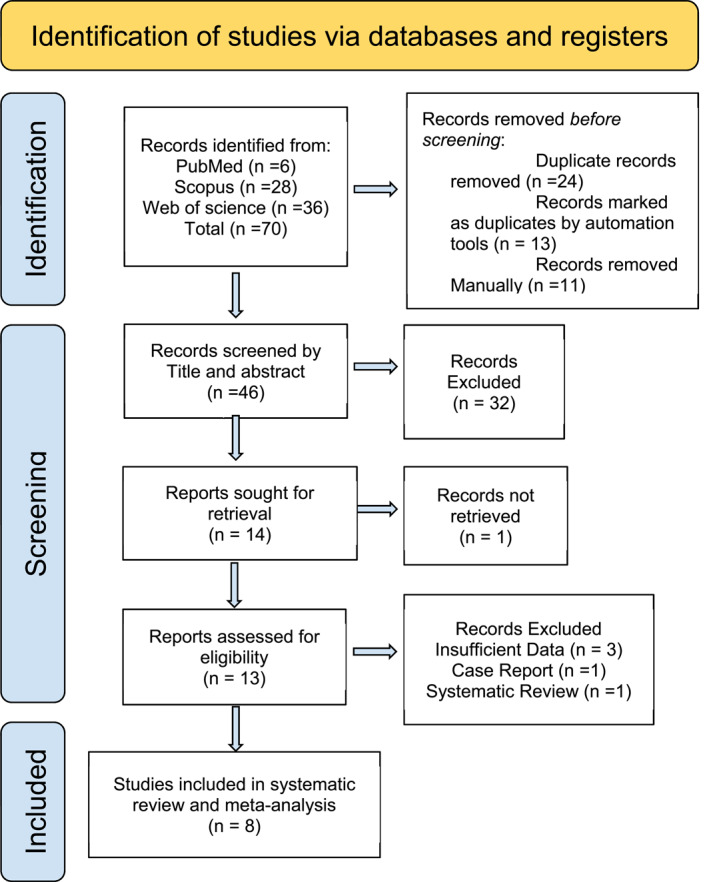
PRISMA 2020 flow diagram for study selection. Flow diagram summarizing the identification, screening, eligibility assessment, and inclusion of studies.

Eight studies met the inclusion criteria [20, 21, 22, 23, 24, 25, 26, 27], encompassing a total of 1107 patients. All included studies included direct comparisons between RA and LA in obese populations. Three studies [22, 24, 27] compared outcomes between obese and non‐obese patients, while another three [21, 22, 25] evaluated differences between LT and PR surgical approaches. The complete study selection process is illustrated in the PRISMA flowchart (Figure 2).

### 3.1 Quality Assessment

Quality assessment using the Newcastle–Ottawa Scale (NOS) indicated high methodological quality in all but one study [24], rated moderate (6 stars) due to limitations in outcome assessment and follow‐up. Three studies [21, 23, 25] scored 9 stars, two [22, 27] scored 8 stars, and one [20] scored 7 stars. One abstract‐only study [26] was not evaluated. Detailed NOS results appear in Table 4.

### 3.2 Robotic versus Laparoscopic Outcomes

**FIGURE 3:**
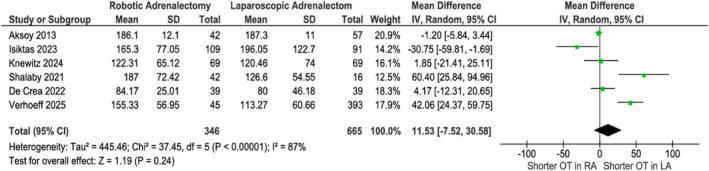
Forest plot of operative time comparing robotic adrenalectomy (RA) versus laparoscopic adrenalectomy (LA) in obese patients.

**FIGURE 4:**
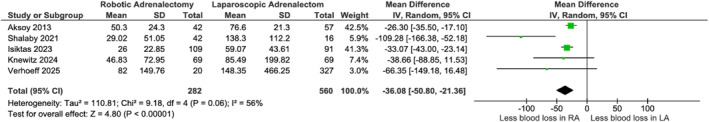
Forest plot of estimated blood loss comparing robotic adrenalectomy (RA) with laparoscopic adrenalectomy (LA) in obese patients.

**FIGURE 5:**
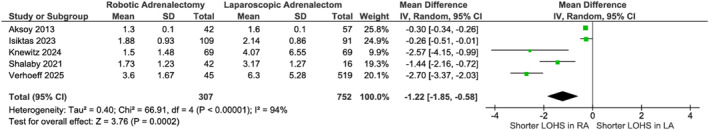
Forest plot of length of hospital stay comparing robotic adrenalectomy (RA) versus laparoscopic adrenalectomy (LA) in obese patients.

**FIGURE 6:**
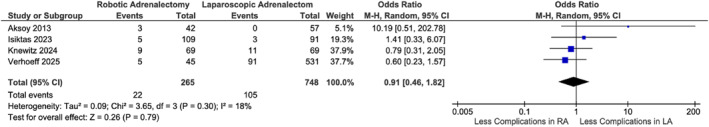
Forest plot complications comparing robotic adrenalectomy (RA) with laparoscopic adrenalectomy (LA) in obese patients.

**FIGURE 7:**
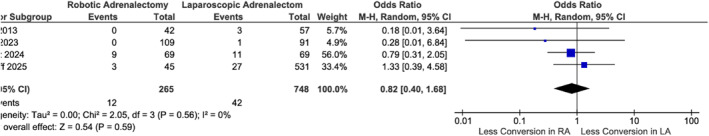
Forest plot of conversion rate comparing robotic adrenalectomy (RA) versus laparoscopic adrenalectomy (LA) in obese patients.

**FIGURE 8:**
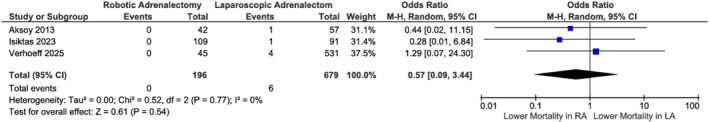
Forest plot of mortality rate comparing robotic adrenalectomy (RA) versus laparoscopic adrenalectomy (LA) in obese patients.

Six studies [20, 21, 22, 23, 25, 26], with 1011 patients, comprising 346 in the robotic arm and 665 in the laparoscopic arm, showed no difference in operative time (*p* = 0.24, Figure 3), and heterogeneity (*I* ^2^ = 87%) decreased to 76% after excluding Verhoeff et al. [22]. RA demonstrated significantly lower blood loss (MD −36 mL, 95% CI [−50.80, −21.36]; *p* < 0.001, Figure 4) in five studies (842 patients, comprising 282 in the robotic arm and 560 in the laparoscopic arm) [20, 21, 22, 25, 26], with heterogeneity (I^2^ = 56%) resolving completely after excluding Shalaby et al. [22]. Hospital stay was shorter for RA (MD −1.22 days, 95% CI [−1.85, −0.58]; *p* < 0.001, Figure 5) in five studies [20, 21, 22, 25, 26] (*n* = 1059), though heterogeneity (I^2^ = 94%) reduced to 83% excluding Verhoeff et al. [20]. No differences were observed in complications (OR 0.91, *p* = 0.79, Figure 6), conversions (OR 0.82, *p* = 0.59, Figure 7), or mortality (OR 0.57, *p* = 0.54, Figure 8). Intra‐ and postoperative complications are shown in detail in Table 5.

**TABLE 5:** Intraoperative and postoperative complications using surgical approach. Reported intraoperative and postoperative complications across robotic, laparoscopic, lateral transabdominal, and posterior retroperitoneal approaches.

Study	Robotic adrenalectomy		Laparoscopic adrenalectomy		Lateral transabdominal		Posterior retroperitoneal	
	Intraoperative	Postoperative	Intraoperative	Postoperative	Intraoperative	Postoperative	Intraoperative	Postoperative
Isiktas et al. (2023) [21]	No intraoperative complications recorded	Urinary tract infection (3) one Pancreatic leak (1) Bowel perforation (1)	Conversion due to inadequate exposure in LT. (1)	Intra‐abdominal Abscess (1) Respiratory Insufficiency (1) Pneumonia (1)	Conversion due to inadequate exposure in LA. (1)	Urinary tract infection (3) one Pancreatic leak (1) Bowel perforation (1)	No intraoperative complications recorded	Pneumonia (1)
Aksoy et al. (2013) [20]	Pneumothorax (1)	Urinary tract infection (1)	Pneumothorax (1)	Prolonged ileus (1)	—	—	—	—
Knewitz et al. (2024) [26]		Grade I (3) Grade II (5) Grade III or IV (3)		Grade I (3) Grade II (6)	—	—	—	—
Verhoeff et al. (2025) [25]	Unspecified (5)	No postoperative complications recorded		Unspecified (4)	Unspecified (5)	Total complications (7)		Total complications (20)
				Both intra‐ and postoperative (11)				
				Unspecified (75)				

### 3.3 Obese versus Non‐Obese Comparisons

**FIGURE 9:**
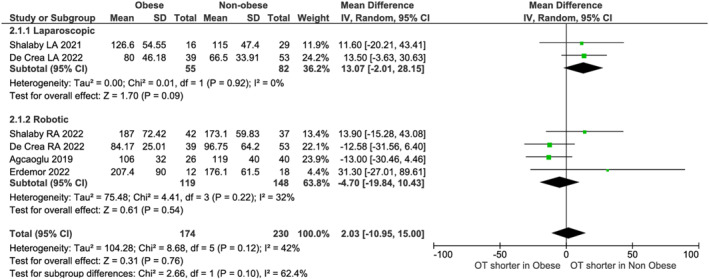
Forest plot comparing operative time between obese and non‐obese patients undergoing laparoscopic (top) or robotic (bottom) adrenalectomy.

**FIGURE 10:**
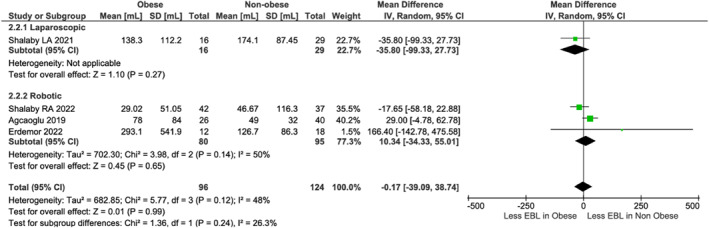
Forest plot comparing estimated blood loss between obese and non‐obese patients undergoing laparoscopic (top) or robotic (bottom) adrenalectomy.

**FIGURE 11:**
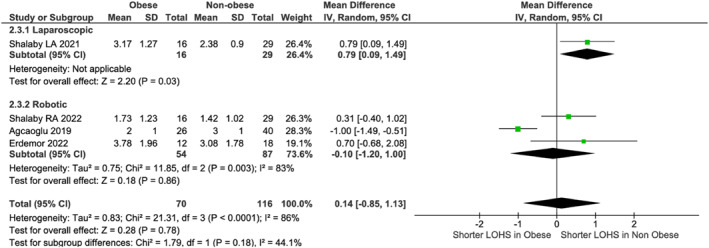
Forest plot comparing length of hospital stay between obese and non‐obese patients undergoing laparoscopic (top) or robotic (bottom) adrenalectomy.

No differences existed in operative time (LA: *p* = 0.09; RA: *p* = 0.22, Figure 9) or blood loss (LA: *p* = 0.27; RA: *p* = 0.65, Figure 10). Non‐obese LA patients had shorter stays (MD: 0.79 days, 95% CI [0.09, 1.49]; *p* = 0.03) in one study [22], though pooled robotic data (three studies [21, 23, 27]) showed no difference (*p* = 0.86, Figure 11). Heterogeneity (*I* ^2^ = 86%) was eliminated (*I* ^2^ = 0%) by excluding Agcaoglu et al. [27].

#### 3.3.1 LT versus PR Approaches

**FIGURE 12:**
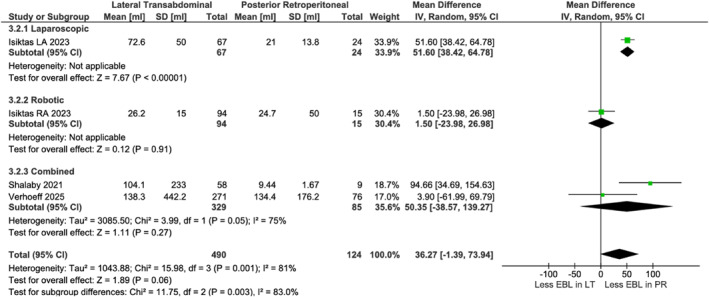
Forest plot comparing estimated blood loss between lateral transabdominal (LT) and posterior retroperitoneal (PR) approaches for adrenalectomy in obese patients. Subgroup analyses were performed for laparoscopic, robotic, and combined approaches.

**FIGURE 13:**
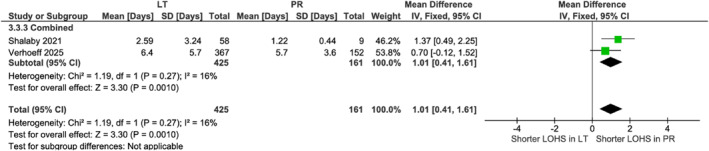
Forest plot comparing the length of hospital stay between lateral transabdominal (LT) and posterior retroperitoneal (PR) approaches for adrenalectomy in obese patients.

**FIGURE 14:**
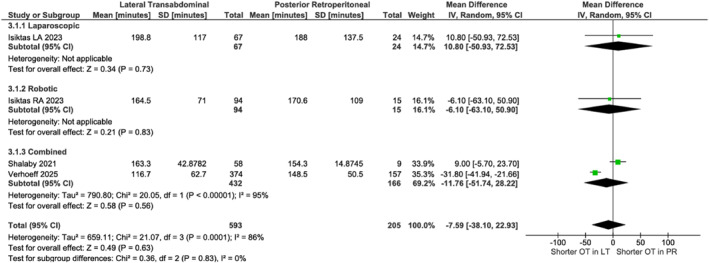
Forest plot comparing operative time between lateral transabdominal (LT) and posterior retroperitoneal (PR) approaches for adrenalectomy in obese patients. Subgroup analyses were performed for laparoscopic, robotic, and combined approaches.

**FIGURE 15:**
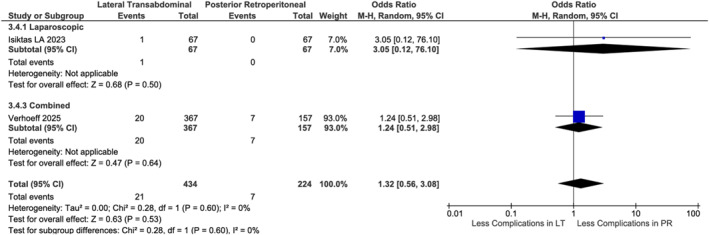
Forest plot comparing complications rate between lateral transabdominal (LT) and posterior retroperitoneal (PR) approaches for adrenalectomy in obese patients.

**FIGURE 16:**
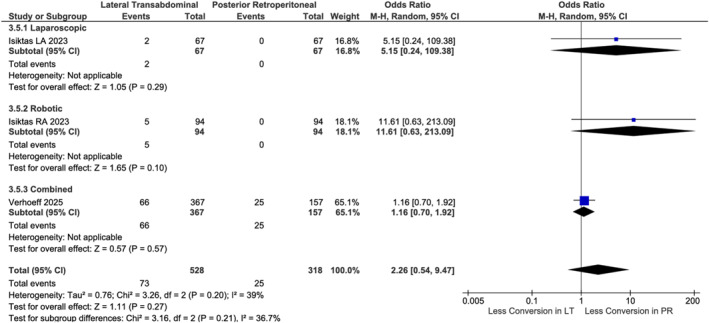
Forest plot comparing conversion to open rates between lateral transabdominal (LT) and posterior retroperitoneal (PR) approaches for adrenalectomy in obese patients.

PR showed lower blood loss in Laparoscopic (MD: 51.60 mL, 95% CI [38.43, 64.78]; *p* < 0.001) [30] but no difference in robotic (*p* = 0.91) or combined analyses (*p* = 0.27, Figure 12). Pooled data [20, 23] (*n* = 586) demonstrated shorter stays with PR (MD 1.01 days, 95% CI [0.41, 1.61]; *p* = 0.001, Figure 13). No differences existed in operative time (*p* = 0.56, Figure 14), complications (*p* = 0.53, Figure 15), or conversions (*p* = 0.27, Figure 16).

RA has shown reduced blood loss and shortened hospital stay compared with LA in obese patients. PR improved blood loss in laparoscopic cases and shortened recovery time compared with LT. Non‐obese LA patients had shorter stays than obese LA patients in one study, but pooled robotic data showed no such difference. No other significant differences were observed.

### 3.4 GRADE Assessment

High‐certainty evidence supported reduced blood loss with RA; moderate certainty favored shorter stays. Operative time evidence was of low certainty due to heterogeneity. Complication, conversion, and mortality outcomes showed moderate certainty with no differences, as per Table 6.

**TABLE 6:** GRADE assessment of the certainty of evidence. Certainty of evidence for the main outcomes comparing robotic and laparoscopic adrenalectomy was assessed using the GRADE approach.

No of studies	Study design	Risk of bias	Certainty assessment		Imprecision	Other considerations	No of patients		Effect		Certainty	Importance
			Inconsistency	Indirectness			RA	LA	Relative (95% CI)	Absolute (95% CI)		
Operative time												
6	Non‐randomised studies	Not serious	Serious	Not serious	Serious	None	346	665	—	MD 11.53 min higher (7.52 lower to 30.58 higher)	⨁⨁◯◯ low	IMPORTANT
Estimated blood loss												
5	Non‐randomised studies	Not serious	Not serious	Not serious	Not serious	None	282	560	—	MD 36.08 mL lower (50.8 lower to 21.36 lower)	⨁⨁⨁⨁ high	CRITICAL
Complications												
4	Non‐randomised studies	Not serious	Not serious	Not serious	Serious	None	22/265 (8.3%)	105/785 (13.4%)	OR 0.91 (0.46–1.82)	11 fewer per 1000 (from 67 fewer to 86 more)	⨁⨁⨁◯ moderate	IMPORTANT
Length of hospital stay												
5	Non‐randomised studies	Not serious	Serious	Not serious	Not serious	None	307	752	—	MD 1.22 days lower (1.85 lower to 0.58 lower)	⨁⨁⨁◯ moderate	IMPORTANT
Mortality												
3	Non‐randomised studies	Not serious	Not serious	Not serious	Serious	None	0/196 (0.0%)	6/679 (0.9%)	OR 0.57 (0.09–3.44)	4 fewer per 1000 (from 8 fewer to 21 more)	⨁⨁⨁◯ moderate	CRITICAL
Conversion rate												
4	Non‐randomised studies	Not serious	Not serious	Not serious	Serious	None	12/265 (4.5%)	42/748 (5.6%)	OR 0.82 (0.40–1.68)	10 fewer per 1000 (from 33 fewer to 35 more)	⨁⨁⨁◯ moderate	IMPORTANT

Abbreviations: CI: confidence interval; MD: mean difference; OR: odds ratio.

## 4 Discussion

**FIGURE 17:**
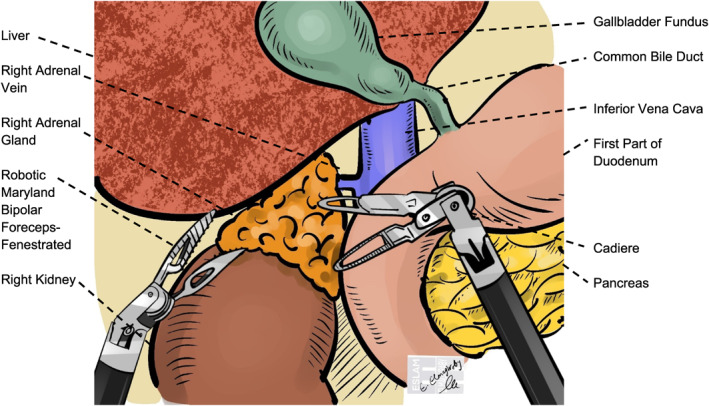
Schematic illustration of a robotic right adrenalectomy. The wristed robotic instruments are shown dissecting the right adrenal gland (orange) from adjacent structures. The critical step depicted is the secure ligation of the short right adrenal vein directly at its junction with the inferior vena cava (blue), a maneuver facilitated by the enhanced dexterity of the robotic platform.

**FIGURE 18:**
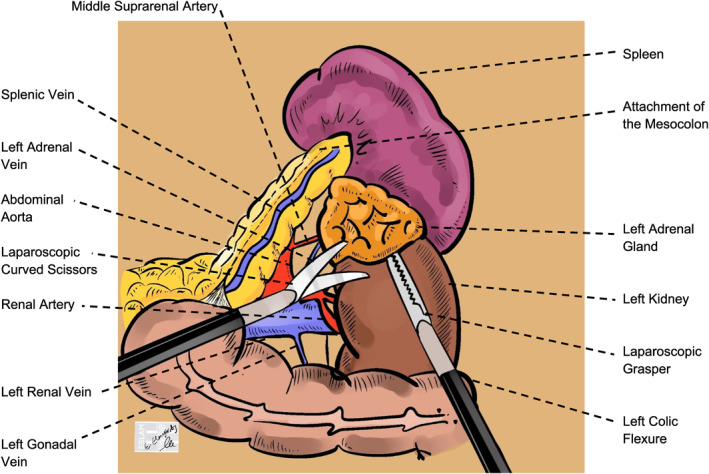
Illustration of a laparoscopic left adrenalectomy. After medial retraction of the spleen (purple) and pancreas tail (yellow) to expose the surgical field, laparoscopic instruments dissect the left adrenal gland (orange). The key action shown is the division of the left adrenal vein after it has been controlled, where it drains into the left renal vein (blue).

Our analysis showed reduced intraoperative blood loss in the robotic approach (Figure 17) compared with the laparoscopic approach (Figure 18), a finding supported by Brandao et al. [31], who attributed this benefit to enhanced precision and visualization. Enhanced instrument precision, motion scaling features, and ergonomics of robotic systems [32] allow for more controlled dissection and improved hemostasis, especially in anatomically complex or vascular regions [31, 33].

Gan et al. found no significant differences; however, both studies combined obese and non‐obese patients or did not specifically account for obesity [34]. Regarding the length of hospital stay, our results indicated a trend towards shorter stays with robotic adrenalectomy. This observation is consistent with Esposito et al., who reported enhanced postoperative recovery in robotic cases [35], while Pineda‐Solís et al. found no substantial differences [36]; although both studies primarily evaluated non‐obese populations, their findings suggest that outcomes in obese and non‐obese patients may be comparable. Shorter hospital stay, in obese patients, optimizes return to work life, due to reduced physical trauma and faster restoration of bodily functions.

In Wang et al., lateral transabdominal and posterior retroperitoneal approaches were pooled, potentially obscuring approach‐specific effects. Our study analyzed them separately and included more patients. Furthermore, while Wang's findings are based solely on U.S. data, limiting applicability to other healthcare systems and patient populations, our SRMA incorporated studies from the USA, Turkey, and Italy, thereby including patients of different ethnicities and surgical care under varied national protocols, enhancing the generalizability of our findings [37]. Complication rates were comparable between the two approaches, as demonstrated by Wang et al. [37], though Isiktas et al. noted a higher rate of minor complications in laparoscopic procedures [21]. Conversion to open surgery was similarly rare for both techniques, as reported by Brandao et al. [31]. Vatansever et al. observed a trend toward higher conversion rates in laparoscopy [38]; however, their analysis combined obese and non‐obese patients, which may confound obesity‐specific effects.

Our analysis showed no significant difference in operative time between robotic and laparoscopic adrenalectomy in obese patients, which aligns with the findings of Brandao et al. [31]. Morino et al. reported shorter operative times for laparoscopic adrenalectomy [39]. Lack of advanced robotic instrumentation and longer institutional experience with the laparoscopic technique contributed to these discrepancies. However, operative time using robotic techniques has markedly decreased over the years [33, 40].

Mortality rates were low and equivalent between robotic and laparoscopic adrenalectomy, with Esposito et al. [35] and Mishra et al. [41], both reporting minimal perioperative deaths. Although Mishra et al. did not report BMI as a baseline characteristic, our findings remain comparable. When comparing obese and non‐obese patients, the only notable difference was a slightly prolonged hospital stay for obese patients undergoing LA, consistent with Rodríguez‐Hermosa et al. [15]. However, Danwang et al. found no such disparity, suggesting that institutional protocols and surgical experience may mitigate the impact of obesity on outcomes of LA [12]. Comparable complication rates between obese and nonobese patients suggest that the technical advantages of robotic surgery—such as enhanced dexterity and three‐dimensional vision [42, 43]—may balance out the anatomical challenges presented by obesity, thus offering an equally safe alternative to laparoscopy when performed by experienced teams.

**FIGURE 19:**
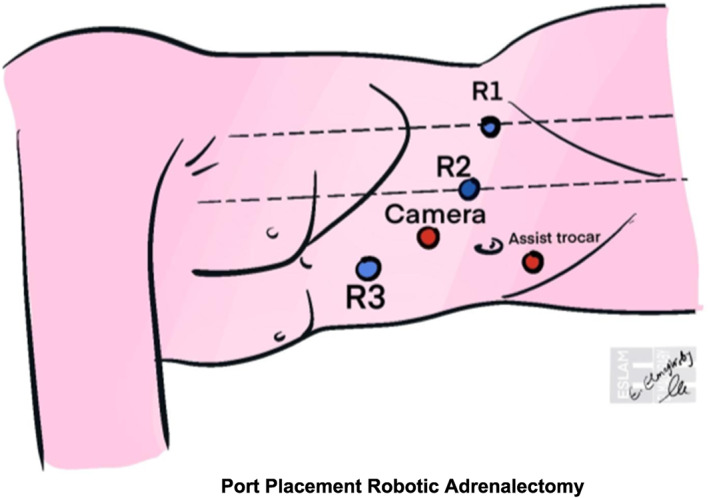
The image illustrates the port placement for a robotic adrenalectomy. The R1 port is designated for the camera, while the R2 port serves as an assist trocar. The patient lies in anterolateral decubitus. The configuration follows a standardized approach for robotic adrenal surgery, ensuring optimal access to the adrenal gland while maintaining triangulation for instrument maneuverability.

**FIGURE 20:**
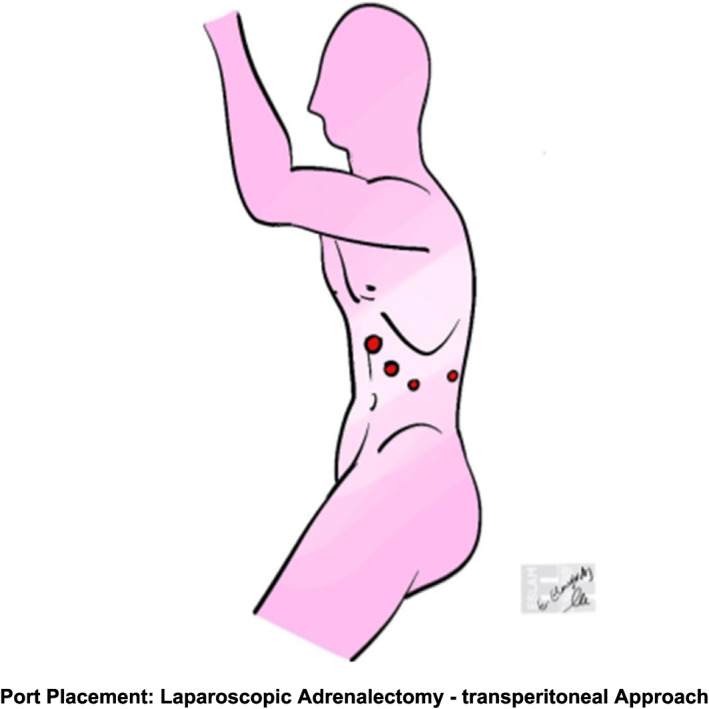
This image shows laparoscopic adrenalectomy port placement. The patient is in the lateral decubitus position. Trocar positions enable medial retraction of organs and adrenal gland exposure. The setup allows division of key structures such as the adrenal vein using conventional laparoscopic tools, ensuring ergonomic access to the left adrenal region while maintaining visibility and instrument mobility.

In evaluating surgical approaches, the posterior retroperitoneal technique (Figure 19) showed reduced blood loss versus the lateral transabdominal technique (Figure 20) in laparoscopic cases [44], likely by avoiding extensive dissection through intraperitoneal fat to access and mobilize the adrenal gland [25]. However, operative times and conversion rates were similar between the LT and PR approaches, consistent with Lee et al. and Barczyński et al. [45]. While some studies, such as Kahramangil et al., reported shorter operative times with the PR, which appeared to result from reduced exposure time [46]. Others, including Yoneda et al. [47] and Naya et al. [48], found no significant differences. Length of stay was generally comparable, though Berber et al. and Kahramangil et al. noted slightly faster recovery with the Laparoscopic retroperitoneal approach [44, 46]. Likely, as PR requires fewer ports and the avoidance of visceral manipulation, both contribute to fewer abdominal adhesions and facilitate recovery [49].

Despite the strengths of our meta‐analysis, several limitations should be acknowledged. The lack of studies with long‐term follow‐up and functional outcome data limits a more comprehensive evaluation of the sustained benefits and potential drawbacks of robotics versus laparoscopic adrenalectomy. Future research should incorporate extended follow‐up durations while also reporting detailed patient histories, including prior abdominal surgeries, and documenting specific complications such as surgical‐site infections, post‐trocar hernias, and tumor recurrence [50]. Moreover, the scarcity of subgroup analyses stratified by tumor pathology and size represents a significant knowledge gap, particularly for obese patient populations.

The observed heterogeneity among studies likely reflects variations in surgical expertise, institutional learning curves, patient complexity, and perioperative protocols. In addition, differences in BMI distribution, the proportion of severely obese patients, and overall cohort composition across the included studies may also have contributed to this variability, particularly for outcomes such as operative time, as supported by Piccoli et al. [32] and Danwang et al. [11]. Most importantly, the predominance of observational studies over randomized controlled trials limits the strength of our conclusions and underscores the need for more rigorous prospective study designs to minimize potential bias.

Although obesity was defined according to the WHO criterion of BMI ≥ 30 kg/m^2^, the severity of obesity varied across the included studies, and this variability could not be differentiated or analyzed separately, which may have introduced additional clinical heterogeneity when interpreting the pooled results.

In addition, robotic adrenalectomy is associated with higher procedural costs and the absence of tactile feedback, which may influence intraoperative decision‐making, particularly in complex or fibrotic cases. These limitations warrant careful patient selection and should be interpreted cautiously, as robotic adrenalectomy is generally associated with higher procedural costs, which may limit its broader adoption despite its perioperative advantages, as highlighted by Ruhle et al. (2019) [31].

Another important limitation of the current literature is the lack of long‐term oncological and functional follow‐up data, which restricts a more comprehensive evaluation of the sustained benefits and potential drawbacks of robotic versus laparoscopic adrenalectomy.

In summary, robotic adrenalectomy may offer advantages in reducing blood loss and potentially shortening hospital stays, but high‐quality prospective studies are needed to confirm these benefits. Both robotic and laparoscopic techniques demonstrate comparable safety and efficacy in obese patients, with outcomes influenced more by surgical expertise and institutional protocols than by inherent differences between the approaches. The choice of technique should be tailored to individual patient factors and the surgeon's experience.

## 5 Conclusion

Robotic adrenalectomy demonstrates advantages over laparoscopic adrenalectomy in obese patients, particularly in reducing intraoperative blood loss and shortening hospital stay, while maintaining comparable operative time, complication rates, and conversion rates. However, subgroup analyses comparing obese and non‐obese patients do not indicate that obesity itself confers a greater relative benefit from the robotic approach; rather, the findings support robotic adrenalectomy as a safe and effective minimally invasive option for obese patients. Further high‐quality prospective studies are warranted to clarify long‐term clinical outcomes and cost‐effectiveness.

## Author Contributions

A.A. created the research idea. A.A., Y.B., M.E., M.E.E., M.I.E., K.M., R.M.L., and F.G. all have participated in designing the research workflow, acquisition and interpretation of data, and Screening of the data. A.A., Y.B., M.E., M.E.E., and F.G. have participated in the writing of the manuscript. A.A., Y.B., and K.M. have analyzed the results. E.E. designed the Figures A.A., Y.B., and K.M. have all revised the whole work. All authors have read and agreed to the final version of the manuscript.

## Funding

The authors have nothing to report.

## Ethics Statement

As this study is a systematic review and meta‐analysis of previously published studies, the PROSPERO registration number is CRD420251102588. All included studies had obtained prior institutional review board (IRB) or ethical approval.

## Patient Consent

The authors have nothing to report.

## Conflicts of Interest

The authors declare no conflicts of interest.

## Permission to Reproduce Material from Other Sources

The authors have nothing to report.

## Supporting information


**Table S1:** PRISMA 2020 checklist.

**Table S2:** AMSTAR 2 methodological appraisal.

## Data Availability

The datasets generated and analyzed during the current study are available from the corresponding author on reasonable request.
