# Supplementary material for: Does Robotic Adrenalectomy Outperform Laparoscopic Approaches in Obese Patients? A Systematic Review and Subgroup Meta‐Analysis of 1,107 Patients
Source: Int J Med Robot. 2026 Apr 20;22:e70169. doi: 10.1002/rcs.70169 (PMC13094886; doi:10.1002/rcs.70169)
Supplement: Supplementary file 2 — Table S2: AMSTAR 2 methodological appraisal. [file RCS-22-e70169-s001.docx]

AMSTAR 2 – Appraisal Tool for Systematic Reviews Including Non-Randomized Studies

This table is designed to facilitate the structured evaluation of systematic review and meta-analysis (SRMA) including non-randomized studies, based on the AMSTAR 2 (A MeaSurement Tool to Assess systematic Reviews, Version 2) instrument.

| No. | AMSTAR 2 Item | Rating (Yes / Partial / No) | Comments |
| --- | --- | --- | --- |
| 1 | Did the research questions and inclusion criteria include the components of PICO? | yes |  |
| 2 | Did the review have an explicit statement that the methods were established prior to the conduct of the review (e.g., a protocol)? | yes |  |
| 3 | Did the review authors use a comprehensive literature search strategy? | yes | MeSH database was used. |
| 4 | Did the review authors perform study selection in duplicate? | yes | Done by 3 reviewers with 4th to resolve conflicts |
| 5 | Did the review authors perform data extraction in duplicate? | yes | 4 reviewers extracted data independently, with 3rd reviewer resolving disagreements. |
| 6 | Did the review authors provide a list of excluded studies and justify the exclusions? | No |  |
| 7 | Did the review authors describe the included studies in adequate detail? | yes |  |
| 8 | Did the review authors use a satisfactory technique for assessing the risk of bias (RoB) in individual studies? | yes | NOS |
| 9 | Did the review authors report on the sources of funding for the studies included in the review? | No |  |
| 10 | If meta-analysis was performed, did the review authors use appropriate methods for statistical combination of results? | yes | DerSimonian McGrath et al  CI |
| 11 | If meta-analysis was performed, did the review authors assess the potential impact of RoB in individual studies on the results? | No |  |
| 12 | Did the review authors account for RoB in individual studies when interpreting/discussing the results of the review? | No |  |
| 13 | Did the review authors provide a satisfactory explanation for, and discussion of, any heterogeneity observed in the results? | yes |  |
| 14 | If they performed quantitative synthesis, did the review authors carry out an adequate investigation of publication bias? | no |  |
| 15 | Did the review authors report any potential sources of conflict of interest, including any funding they received for conducting the review? | yes | No funding |
| 16 | Were the review authors’ conclusions supported by the results and did they consider the limitations of the included studies? | yes | GRADE |

**Reference: DOI:**[**10.1136/bmj.j4008**](https://doi.org/10.1136/bmj.j4008)

**Supplementary Table 2. AMSTAR 2 methodological appraisal.**
AMSTAR 2 assessment of the methodological quality of the present systematic review and meta-analysis, including ratings for each critical and non-critical domain with accompanying explanatory comments.
